# A Broken Metallic Tracheostomy Tube

**Published:** 2012

**Authors:** Mohammad Naeimi, Mohsen Rajati, Tirzad Fooladvand

**Affiliations:** 1 *Ear, Nose, Throat, Head and Neck surgery Research Center, Mashhad University of Medical Sciences, Mashhad, Iran*; 2 *Department of otorhinolaryngology, Qhaem Hospital, Faculty of Medicine, Mashhad University of Medical Sciences, Mashhad, Iran.*

Tracheostomy is an upper airway bypass which is life saving in certain occasions. However, there are several early and late complications. One of these rare mishaps is the broken cannula, few cases of which has been reported so far. Herein we are reporting a rare case of broken tube as a late complication of indwelling tracheostomy tube. A 52 year old woman with moderate respiratory distress was brought to the otolaryngology emergency ward. The patient’s past history included 2 thyroid surgeries for papillary carcinoma 3 years earlier and since then she was dependant on the tracheotomy tube. She had been using the metallic double lumen tube and she did frequently take the tube out (the outer cannula!), clean it and put it back in place. About 2 hours before admission she felt difficult air passage due to the dried secretions and she had tried to take the tube out but it broke and only the neckplate came out with the cannula remaining in the trachea. X-ray was taken which confirmed the patient’s statement. 

**Fig. 1:**
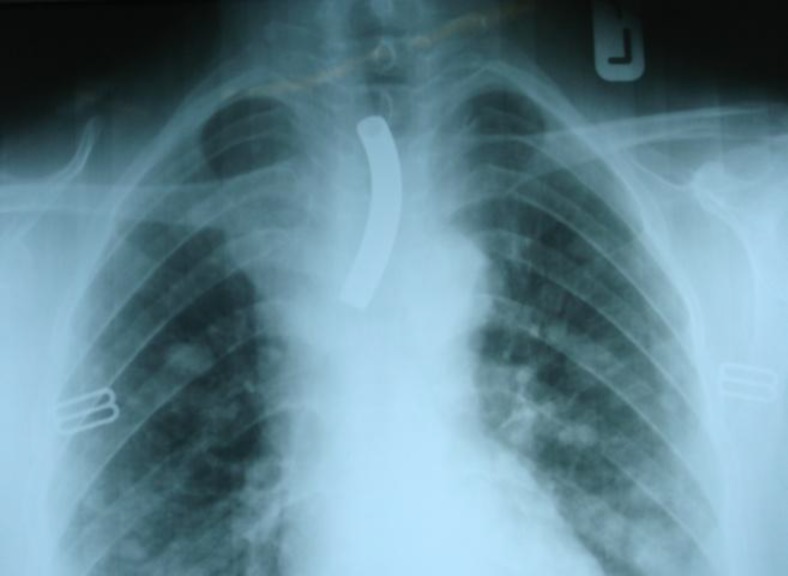
Chest-X-ray showing the metallic cannula within the trachea

The patient was admitted in the operating room and under systemic intravenous sedation the tube which had moved a bit deeper then, was taken out using long forceps. The patient was discharged the day after (fig.1).

